# Reward and reinforcement activity in the nucleus accumbens during learning

**DOI:** 10.3389/fnbeh.2014.00114

**Published:** 2014-04-03

**Authors:** John T. Gale, Donald C. Shields, Yumiko Ishizawa, Emad N. Eskandar

**Affiliations:** ^1^Nayef Al-Rodhan Laboratories for Cellular Neurosurgery and Neurosurgical Technology, Department of Neurosurgery, Harvard Medical School, Massachusetts General HospitalBoston, MA, USA; ^2^Department of Anesthesia, Critical Care and Pain Medicine, Harvard Medical School, Massachusetts General HospitalBoston, MA, USA

**Keywords:** nucleus accumbens, learning, reward, incentive salience, operant conditioning

## Abstract

The nucleus accumbens core (NAcc) has been implicated in learning associations between sensory cues and profitable motor responses. However, the precise mechanisms that underlie these functions remain unclear. We recorded single-neuron activity from the NAcc of primates trained to perform a visual-motor associative learning task. During learning, we found two distinct classes of NAcc neurons. The first class demonstrated progressive increases in firing rates at the go-cue, feedback/tone and reward epochs of the task, as novel associations were learned. This suggests that these neurons may play a role in the exploitation of rewarding behaviors. In contrast, the second class exhibited attenuated firing rates, but only at the reward epoch of the task. These findings suggest that some NAcc neurons play a role in reward-based reinforcement during learning.

## Introduction

The process of associative learning, whereby the brain links sensory stimuli with specific motor behaviors and expected rewards, is fundamental to adaptation and survival. Evidence suggests that a critical portion of this process is encoded in the nucleus accumbens core (NAcc) and is in part mediated through the actions of the neurotransmitter dopamine (Schultz, 1998, 2000; Ikemoto and Panksepp, 1999; Bar-Gad et al., 2003; Wise, 2004; Graybiel, 2005; Frank and O'Reilly, 2006; Daniel and Pollmann, 2010) although the precise role of dopamine in this process is a source of considerable debate (Salamone et al., 2005).

Anatomical, neurochemical, and brain lesion data suggest that the NAcc plays a role in modulating the motivation to perform reward-oriented behaviors as a “limbic-motor interface” (Mogenson et al., 1980). The NAcc receives glutamatergic inputs from orbitofrontal/prefrontal cortex, basolateral amygdala, and hippocampus (areas involved with stimulus properties, preferences, and memories), while dopaminergic input is received from ventral tegmental area neurons (Poletti and Creswell, 1977; Beckstead, 1979; Russchen et al., 1985; Selemon and Goldman-Rakic, 1985; Haber et al., 1990; Brog et al., 1993; Wright and Groenewegen, 1995; Fudge and Haber, 2002). NAcc outputs include projections to the ventral pallidum, the dorsomedial thalamus (which projects back to the orbitofrontal cortex), pedunculopontine tegmentum, and a significant projection to dopaminergic areas of the midbrain (Groenewegen and Russchen, 1984; Haber et al., 1990; Heimer et al., 1991; Nicola et al., 2000; Zahm, 2000; Wise, 2004). Thus, the NAcc is positioned to receive diverse information from brain regions believed to encode aspects of reward-related information, while its projections can modulate nuclei associated with generation of motor behaviors and dopamine release (Joel and Weiner, 2000; Sesack and Grace, 2010).

Lesion and drug studies have demonstrated that disruption of the NAcc results in decreased goal-directed behavior, dysfunction of reward encoding and learning as well as reduction in locomotor and approach behaviors (Wise et al., 1978a,b; DiCiano et al., 2001; Parkinson et al., 2002; Wise, 2004; Day and Carelli, 2007). Correspondingly, dysregulation of the NAcc has been implicated in a number of disease states including major depression, drug addiction, and Parkinsons disease (Deutch, 1993; Gao et al., 2003; Giacobbe and Kennedy, 2006). One potential explanation for the above findings, the “incentive salience” hypothesis, posits that dopamine signaling via the mesolimbic dopaminergic pathway (which partially includes the NAcc) regulates motivation by associating values with environmental stimuli that predict reward (Berridge and Robinson, 1998; McClure et al., 2003a,b; Wise, 2004; Salamone et al., 2005). If this hypothesis is correct, the assignment of predictive value should be updated with operant conditioning, whereby the associated value placed on a stimulus is low before learning and progressively increased as a particular association is mastered.

Moreover, during classical conditioning, the repetitive pairing of an external stimulus (e.g., visual, auditory, tactile) with a reward prompts increased firing rates of NAcc phasically active neurons (PAN's) during stimulus presentation (Schultz et al., 1992). In contrast, when rewards are omitted, following previously conditioned stimuli (extinction), firing rates attenuate during stimulus presentation. Unlike the reflexive responses of classical conditioning, operant conditioning requires formation of associations between external stimuli and spontaneously generated, volitional behaviors that result in reward. Furthermore, the mechanisms that promote reinforcement of profitable associations and attenuation of unprofitable associations in operant conditioning remain poorly understood. Prior studies suggest that the process of reinforcement and attenuation of behavior is governed by convergent interactions between striatal tonically active neurons (TAN's) that convey information regarding outcomes and midbrain dopaminergic neurons that encode information specific to reward prediction (Morris et al., 2004).

Thus, we examined the activity of NAcc neurons in non-human primates as they performed a visual-motor associative learning task wherein they focused on a central point on the screen until an object appeared (Stimulus). After a variable delay, the fixation point disappeared (GoCue), at which point the monkey was required to make a saccade from the center of the screen to one of four targets (Movement). An auditory tone (Feedback/tone) and color change of the selected target indicated whether the animal made the correct or incorrect choice. The former was followed by juice administration (Reward). We found that during learning, responsive neurons can be divided into at least two distinct classes. The first class of neurons (Class I) exhibited a progressive increase in activity that was then maintained after novel visual-motor associations were mastered. These learning-related increases in activity were observed at the go-cue, feedback/tone and reward epochs of the behavioral task, suggesting a role in exploiting learned rewarded behaviors. In contrast, the second class of neurons demonstrated a decrease in activity that occurred only during the reward periods of the task. Hence, these “Class II” neurons may be involved in encoding profitable associations via down regulation of neuronal activity (Krause et al., 2010; Jurado-Parras et al., 2012). Therefore, these distinct activity patterns suggest NAcc neurons interact to process reward information, and subsequently provide a graded motivational signal as associations are learned.

## Results

### Visual-motor association task

**Figure 1:**
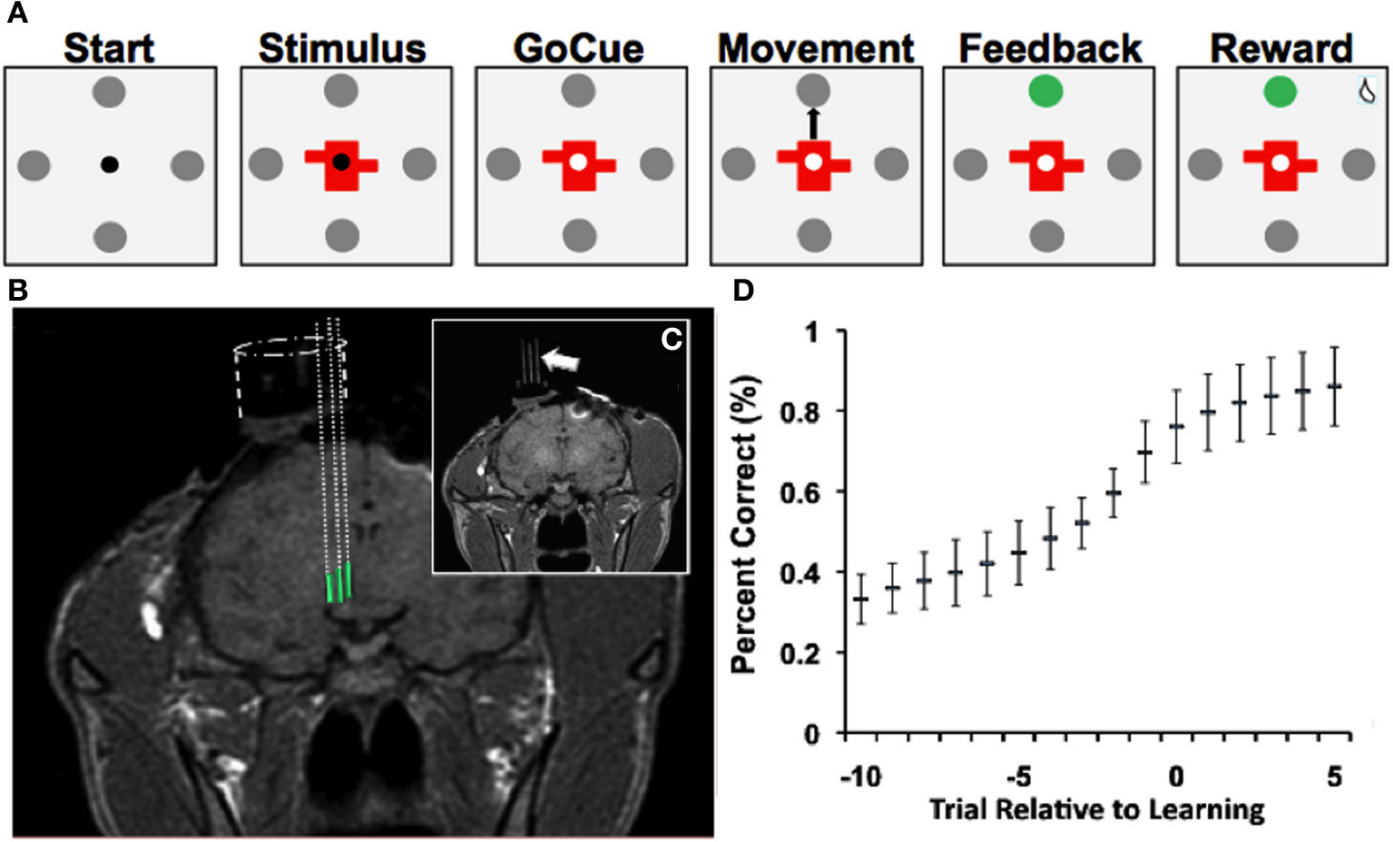
**Learning task, recordings and learning. (A)** Depiction of visual-motor learning task. Each trial began (“Start”) with the presentation of a central fixation point (0.2° diameter) surrounded by four gray targets (1° diameter and 10° from the center). Animals were required to fixate within 2° of the fixation point for 500 ms. Then either a novel or familiar stimulus appeared for 500 ms at the center, with the fixation point still visible (“Stimulus”). After a variable delay of 500–1000 ms, the fixation point disappeared (“Go-Cue”), at which point the monkey was allowed to saccade from the center of the screen to one of the four targets (“Movement”). Once the animal fixated on a target for 500 ms, an auditory tone (“Feedback/Tone”) and a color change of the selected target indicated whether the animal made the correct (high pitch) or incorrect (low pitch) choice. A correct choice was followed by a juice reward after an additional 500 ms delay (“Reward”). An incorrect choice was followed by no juice reward. If at any point the animal failed to meet these criteria, the trial aborted and no reward was given. Trials were separated by a 1250–2250 ms interval. **(B)** T1-weighted MR images demonstrate electrode trajectories for recordings in the NAcc (green). **(C)** Trajectories were confirmed with placement of fiducial rods (arrow). **(D)** Behavioral performance (“learning curve”) on the visual-motor association task (mean ± SD; Bernoulli estimate of performance). The x-axis represents the trial relative to learning, and the y-axis represents the percent correct.

Two adult male rhesus monkeys (*Macaca mulatta*) were used in this study in accordance with NIH and Massachusetts General Hospital Animal Research guidelines. The visual-motor association task required the animals to view objects presented on a screen and then make a saccade to one of four targets (Figure 1A). The animals learned, by trial-and-error, to associate each specific novel geometric visual stimulus with a unique eye movement to one of the four targets. Eye position was monitored with an infrared video eye-tracking system (ISCAN Inc.; Woburn, MA) that provides eye coordinates to the behavioral control software (MonkeyLogic, www.monkeylogic.net).

Each trial began with the presentation of a central fixation point (0.2° diameter) surrounded by four gray targets (1° diameter and 10° from the center) (Figure 1A, “Start”). Animals were required to fixate within 2° of the fixation point for 500 ms. Then either a novel or familiar stimulus appeared for 500 ms at the center, with the fixation point still visible (Figure 1A, “*Stimulus*”). After a variable delay of 500–1000 ms, the fixation point disappeared (Figure 1A, “*GoCue*”), at which point the monkey was required to make a saccade from the center of the screen to one of the four targets (Figure 1A, “*Movement*”). Once the animal fixated on a target for 500 ms, an auditory tone (Figure 1A, “*Feedback/Tone*”) and a color change of the selected target indicated whether the animal made the correct or incorrect choice. A correct choice was followed by a juice reward after an additional 500 ms delay (Figure 1A, “*Reward*”). An incorrect choice was followed by no reward. If at any point the animal failed to meet these criteria, the trial was aborted, and no reward was given. Trials were separated by a 1250–2250 ms interval.

During each learning block, two novel stimuli (randomly generated geometric objects) and two familiar stimuli (randomly selected from a group of well-trained familiar objects with established movement directions) were presented. Each visual stimulus was associated with a unique saccade direction. The use of the familiar objects served two important functions. First, familiar trials provide an impetus for the animals to continue working during the initial phase of learning, when correct choices for novel objects occur at a low frequency. Second, the familiar object trials provide an important control since neuronal activity for familiar objects does not depend on learning.

Once the animals performed 16 correct trials for each object, the novel stimuli were replaced by two new randomly generated novel stimuli. This process was repeated multiple times for each neuron recorded, such that numerous instances of visual-motor associative learning were recorded for each neuron. Familiar and novel object trials were pseudo-randomly interleaved (i.e., each objects was randomly presented before any were repeated) within each block. Animals were trained on the behavioral task until they learned a minimum of four learning blocks per learning session. After behavioral training, the animals were implanted with recording chambers (Figure 1B) so that single-unit recordings could be obtained from the NAcc as the animals performed the task (see Experimental Procedures, “Single-Unit Recording and Localization of NAcc”).

### Learning rates and neuronal database

During the study, animals successfully learned 64% (*n* = 558/878) of novel object associations (to a 99% confidence interval) during the visual-motor association task and learned 4.7 ± 0.3 (mean ± s.e.m.) novel objects per recording session. On average, the animals learned novel associations in 10.0 ± 0.3 trials (mean ± s.e.m.; counting preceding incorrect and correct trials). Behavioral performance of the task (Figure 1D) demonstrated that the animals' performance started near chance (25%) and reached approximately 80% after learning occurred. Among familiar objects presented, animals selected the correct target in 98% of trials. Moreover, reaction times during the task were correlated with behavioral performance (*p* < 0.001; linear regression). That is, as the animals learned new associations, the time needed to initiate movement decreased.

A total of 132 neurons were recorded from the NAcc from two non-human primates (monkey 1, *n* = 86; monkey 2, *n* = 46) as the animals performed the visual-motor association task. Of the 132 neurons recorded during the task, 88 (67%) were determined to be task responsive, and were further analyzed. The remaining neurons (*n* = 44/132) were classified as non-responsive and excluded from subsequent analysis. The aggregate median baseline firing rates (at the start of the trial) for task responsive neurons were 7 spikes/second (4–16 spikes/second quartiles; Table 1). Baseline median firing rates between Class I [6.9 spikes/second (4–13)] and Class II [7.1 spikes/second (4–20)] neurons were not significantly different (Mann-Whitney; *p* = 0.5). In addition, the aggregate mean discharge rate (Table 1) of responsive neurons demonstrated a significant increase in activity during the go-cue, feedback/tone, and reward epochs of the task (Friedman analysis of variance; *p* < 0.001, Dunn's correction). However, at the individual neuron level, significant differences emerged between the two classes of neurons during learning.

**Table 1:** **Percent modulation and firing rate [spikes/second; median (quartiles)] of responsive neurons in familiar trials by task epoch**.

**Group**	***N***	**Start**	**Stimulus**	**GoCue**	**Tone**	**Reward**
All	88	6%; 7.0 (4–16)	10%; 8.0 (4–17)	25%; 9.8 (5–19)	22%; 9.7 (5–19)	28%; 9.7 (5–17)
Class I	39	3%; 6.9 (4–13)	11%; 8.0 (3–14)	21%; 10.1 (5–19)	21%; 9.6 (5–19)	30%; 11.1 (6–19)
Class II	49	8%; 7.1 (4–20)	11%; 8.0 (5–20)	30%; 9.5 (5–19)	25%; 9.9 (5–19)	30%; 9.0 (5–17)

### Modulation relative to learning

In order to evaluate neuronal activity in relation to learning, the series of correct and incorrect responses for each novel object was analyzed using a state-space approach to establish the trial at which an animal reached the learning criterion for a particular novel visual stimulus (Wirth et al., 2003; Williams and Eskandar, 2006; Sheth et al., 2011). This analysis approach provides the trial number (criterion trial) at which the animal's choice was statistically greater than chance at a 99% confidence interval. The criterion trial was then used to align responses to novel object trials from 10 trials before to five trials after the criterion trial.

Responsive neurons were pooled into two groups based upon their correlation with the learning curve during the reward period of the task. Briefly, neurons that demonstrated a positive correlation between firing rates to novel objects and the learning curve during the reward period were separated into one group (Class I) and those that demonstrated a negative correlation were pooled into a second group (Class II).

### Response to familiar objects

Familiar object trials do not require learning. These visual cues and their associated movement directions were presented to the animals thousands of times during training and were extremely well learned by the time of the experiment. Firing rate modulation during familiar trials demonstrated different patterns of activity between the two groups of responsive neurons. The population of Class I neurons (39 of 88 responsive neurons, 44%), responded to the behavioral task by a consistent increase in firing rate, compared to baseline, during the go-cue, feedback/tone and reward periods of the task (Table 1, Friedman analysis of variance; *p* < 0.001, Dunn's correction). In contrast, the population firing rate of Class II neurons (56%, 49 of 88 responsive neurons) did not demonstrate a significant change (Table 1).

### Response to novel objects

**Figure 2:**
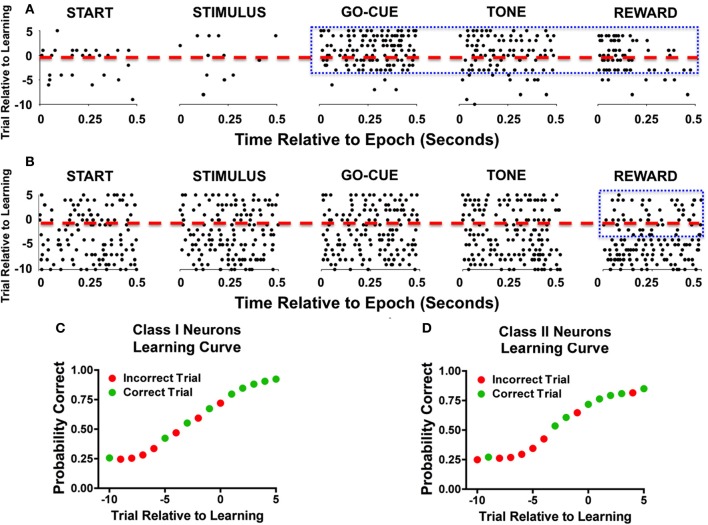
**Representative examples of individual neurons during learning. (A)** Raster illustration of a Class I neuron during a single learning event. The y-axis represents the trial number relative to learning (dashed red line represents criterion trial), and the x-axis represents the time relative to the start of the epoch. As seen during the go-cue, feedback/tone, and reward periods there is an increase in activity (enclosed blue box). **(B)** A representative example of a Class II neuron during a single learning event. As seen in this example, the neuronal firing rate decreases during the reward period as learning occurs (enclosed blue box). **(C)** Behavior performance of the Class I neurons during the learning event. The green dots represent correct trials and the red dots depict incorrect trials. The y-axis represents the Bernoulli estimate of performance, and the x-axis represents the trial relative to learning. **(D)** Behavioral performance of Class II neurons during the single learning event.

Analysis of activity during novel object trials also revealed significant differences between the two classes of responsive neurons. The learning-related activity of Class I and II neurons can be appreciated as representative neurons during a single learning event (Figure 2). The raster plots of a Class I neuron (Figure 2A) demonstrate a significant increase in activity in trials during and after learning at the go-cue, feedback/tone, and reward epoch of the task (Figure 2A, enclosed box). In contrast, a Class II neuron (Figure 2B) had consistent discharge rates in all epochs of the task except for the reward period, during which it exhibited a decrease in activity near the learning criterion and afterward (Figure 2B, enclosed box). The learning curves for both representative neurons started at approximately 25% (or chance) before learning, and increased to greater than 70% after learning had occurred (Figures 2C,D).

**Figure 3:**
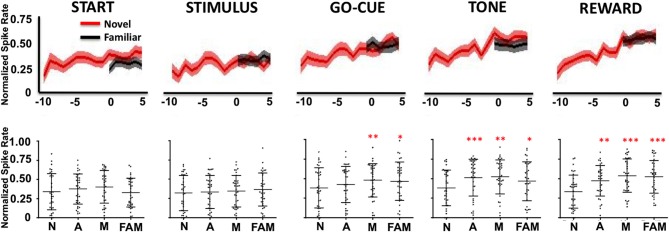
**Neuronal firing rates for Class I neurons (*n* = 39) relative to learning**. The upper row illustrates the average normalized firing rate (mean ± s.e.m. spikes per second; y-axis) of Class I neurons from ten trials (*t* = −10) before, to five trials (*t* = 5) after learning for novel objects (red lines) and for familiar objects (black lines). The column labels represent the epoch (e.g., Start, Stimulus, etc.). The lower row depicts the average normalized firing rate (mean ± SD) for each neuron (black dots) for familiar (FAM) and for novel objects when animals are naive (N; trials −10 to −7), acquiring (A; trials −1 to 1) and after they have mastered (M; trials 3–5) the novel association (^*^p < 0.05, ^**^p < 0.01, and ^***^p < 0.001; Friedman repeated measures analysis of variance with Dunn's correction).

As a population, Class I neurons demonstrated a significant gradual increase in firing rates during the stimulus, go-cue, feedback/tone, and reward periods of the task as learning occurred (Figure 3). Neuronal activity prior to learning (trials, −10 to −7) was significantly lower than activity for familiar objects trials (Figure 3 lower panels; at comparable epochs; repeated measures Freidman Analysis with multiple comparisons correction; *X*^2^_Go(3)_ = 10.87, *X*^2^_Tone(3)_ = 14.81, *X*^2^_Reward(3)_ = 38.15, ^*^p < 0.05, ^**^p < 0.01, and ^***^p < 0.001). However, after learning (trials 3–5), novel object-related activity significantly increased and matched the activity for familiar object trials. Importantly, the increases in activity in the feedback/tone and reward periods occurred prior to significant changes in the go-cue period [Figure 3; statistical significance was reached at learning (trials −1 to 1)].

**Figure 4:**
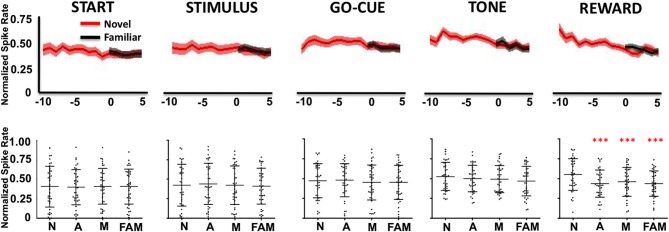
**Neuronal firing rates for Class II neurons (*n* = 49) relative to learning**. (Labels are the same as described in Figure 3).

In contrast, Class II neurons, as a population, exhibited a decrease in activity during the reward period of the task (Figure 4). Like the Class I neurons, the novel object-related activity in the reward epoch before learning was significantly different than familiar object activity, and was significantly different from novel activity at or after learning (Figure 4, reward period: repeated measures Freidman Analysis; *X*^2^_Reward(3)_ = 28.66, ^***^p < 0.001).

### Neuronal responses to correct and incorrect trials

**Figure 5:**
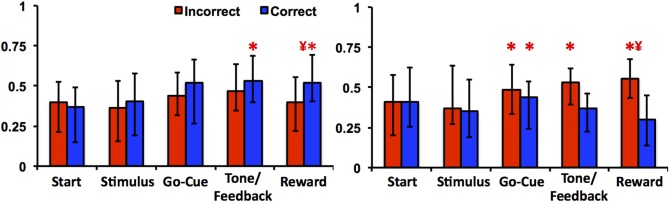
**Neuronal activities of Class I and Class II neurons during incorrect and correct novel object trials**. The left panel illustrates the average firing rate (median ± quartiles; normalized spikes per second) of Class I neurons compared to baseline (mean firing rate at the start epoch) in incorrect (red) and correct (blue) trials. The right panel illustrates the average firing rate of Class II neurons (“^*^” denotes statistical significance relative to baseline rates, and “**¥**” denotes difference between correct and incorrect firing rates within the same epoch; paired Friedman Analysis of Variance with Dunn's correction, *p* < 0.05).

The learning-related patterns of activity for Class I and II neurons can also be demonstrated by examining their firing rates relative to correct and incorrect trials. As illustrated in Figure 5, Class I neurons exhibited a significant increase in firing rates in correct trials at feedback/tone and reward epochs of the task [repeated measures Freidman Analysis with multiple comparisons correction; *X*^2^_(10)_ = 64.39, p < 0.05] compared to baseline rates (i.e., start epoch). Firing rates were also greater between correct than for incorrect trials in reward epoch. This suggests that the learning-related changes in neuronal activity are robust because comparisons between correct and incorrect trials are only a crude measure of learning (i.e., incorrect trials generally occur before learning while correct trials occur more often after learning). Of note, this analysis independently confirms the previously described learning results, as it does not require an algorithm to define when learning occurred (e.g., the learning criterion). In essence, the activity changes of Class I neurons were more gradual over the course of the learning analysis; thus, the activity of the Class I neurons appears to follow the change in reward prediction (i.e., the learning curve).

In contrast, the Class II neurons demonstrated a significant increase in activity during incorrect trials in the reward epoch of the task (Figure 5). Relative to baseline firing rates, neuronal activities for the go-cue (correct and incorrect trials), feedback/tone (incorrect trials) and reward period (incorrect trials) were significantly different [repeated measures Freidman Analysis with multiple comparisons correction; *X*^2^_(10)_ = 63.8, p < 0.05]. Moreover, the absolute change in firing rate for the Class II neurons was greater in this analysis than was demonstrated by the learning analysis; thus appears that the activity of the Class II neurons encodes information with respect to immediate trial outcome (reward or no reward) independent of previous trials (Morris et al., 2004).

### Neuronal responses to correct and incorrect trials relative to learning

**Figure 6:**
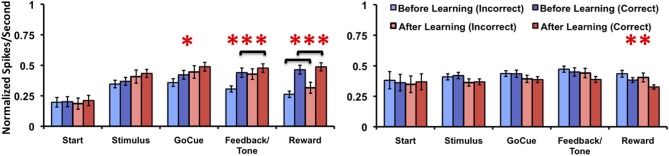
**Neuronal activities of Class I and Class II neurons during incorrect and correct novel object trials relative to learning**. Illustration of the mean normalized firing rates for incorrect trials before (light blue), correct trials before (blue), incorrect trials after (light red), and correct trials after (red) learning for Class I (left panel) and Class II neurons (right panel). (difference in learning factor in matched 2-Way analysis of variance, ^*^p = 0.03, ^**^p = 0.01, ^***^p < 0.001; black brackets denote *post-hoc* statistical difference with Bonferroni correction, *p* < 0.01; error bars are ± s.e.m.)

Since the animals sometimes performed correctly before learning the task and incorrectly on trails after learning occurred, it is unclear how these responses affected analysis of the firing rates with inclusion of trials just before and after the learning criterion. To account for this we compared the firing rates of incorrect trials to the correct trials relative to learning. In this comparison, Class I neurons (Figure 6, left panel) demonstrated a significant difference in firing rates only for the factor of learning at the go-cue [*p* = 0.03; *F*_(1, 70)_ = 5.1], feedback/tone [*p* < 0.001; *F*_(1, 70)_ = 10.9] and reward [*p* < 0.001; *F*_(1, 70)_ = 23.2] epochs of the task (matched sample 2-Way analysis of variance for each epoch). The statistical analysis failed to find significant difference for either the factor of correctness or the interaction. *Post-hoc* analysis revealed a significant increase in firing rates between correct trials before and after learning during the feedback/tone epoch and for both correct and incorrect trials before and after learning during the reward epoch (*p* < 0.01, Bonferroni correction). In contrast, Class II neurons (Figure 6, right panel) demonstrated a significant difference in firing rates only for the factor of learning at the reward [*F*_(1, 84)_ = 6.2] epoch of the task (matched sample 2-Way analysis of variance for each epoch). *Post-hoc* analysis failed to identify differences between correct and incorrect trials before and after learning (*p* < 0.05, Bonferroni correction). These results are consistent with the prior analysis, where activities were compared relative to learning.

Of note, there were cases after the animal learned the association (as defined by the learning criterion) where no incorrect responses were made. As such, these neurons where not used in the subsequent analysis. This was the case for three neurons in the Class I group and six neurons in the Class II group. Moreover, caution must be taken when interpreting the *post-hoc* analysis data due to the unbalanced number of samples for correct trials before and after learning (the same is true for incorrect trials).

### Receiver operator characteristic of neuronal responses

**Figure 7:**
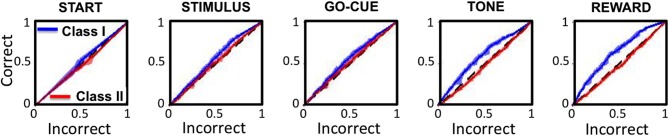
**Receiver operating characteristics (ROC curves) for Class I (blue traces) and Class II (red traces) neurons for each epoch of the task**. The x- and y-axes represent the sensitivity of the classifier for a correct and incorrect choice on the subsequent trial, respectively. The black dashed lines represent unity (or chance).

Receiver operator characteristic (ROC) analysis was performed on the activity of the Class I and II neurons to test the sensitivity of responsive neurons in predicting behavior in subsequent trials. The ROC analysis for Class I neurons (Figure 7, blue traces) demonstrated a significant positive deviation from unity (black dashed lines) for every epoch of the task except for the start of the trial. In contrast, the Class II neurons (Figure 7, red traces**)** significantly deviate from unity only at the reward epoch of the task.

These analyses indicate that increased neuronal activity in Class I neurons predicts that the animal will likely make the same “correct” choice on a subsequent presentation of the same visual cue, even when there are other intervening trial types. For these neurons, the correct choice prediction is statistically significant starting at the presentation of the stimuli, and becomes increasingly more significant with each sequential epoch. In contrast, the Class II neurons only predict behavior on subsequent trials at the reward period of the task, after the behavior had been completed.

### Controls

For both classes of neurons, firing rates during familiar object trials were stable (Figures 4, 5, Upper panels; Black lines) and did not change during the recording blocks. The familiar object trials serve as an important control because no learning occurs during these trials. Thus, activity changes relative to novel object learning can be dissociated from systematic experimental confounds such as neuronal drift, global changes in arousal, or changes in satiety.

Novel object firing rates for Class I neurons were lower than that of familiar object rates before learning (at the go-cue, feedback/tone, and reward periods) and eventually matched firing rates of familiar object trials as learning occurred. Likewise, novel object firing rates for Class II neurons during the reward period were greater than those of familiar object trials, and decreased to familiar object rates as learning occurred. Therefore, changes in novel object neuronal activity can be attributed to the effects of learning rather than other sources. Moreover, neither class of neurons demonstrated any deviation from unity during the start epoch. This also serves as an important control, since the animal has no information about the trial, and a significant deviation from unity may indicate the animal's bias toward a given stimulus or direction.

## Discussion

Incentive salience is characterized by the concept that motivation is governed by associating values with reward-predicting stimuli. The associated values are thought to qualitatively represent a degree of “wanting” rather than “liking” (or hedonic phenomena); thus, presentation of the associated stimuli is transformed into reward expectation that ultimately drives goal-oriented behaviors (Robinson and Berridge, 1993). In addition to its putative role in incentive salience during normal behavior, the NAcc has been studied extensively in relation to the pathophysiology of addiction and depression (Robinson and Berridge, 1993; Pizzagalli et al., 2009). Prior electrophysiological studies have demonstrated that NAcc activity reflects the expectation of upcoming rewards (Schultz et al., 1992; Schultz, 1998; Knutson et al., 2001; McClure et al., 2003a,b), and encodes the anticipated reward value of conditioned stimuli, whereby NAcc activity is greater when high rewards are expected (Simmons et al., 2007).

In the current study, we observed two distinct populations of neurons that exhibited characteristic patterns of neuronal activity in relation to learning. Class I neurons demonstrated gradually increased activity during the go-cue, feedback, and reward periods of the task. Of note, these neurons demonstrated increased activity before the execution of the behavior (at the go-cue), which suggests that they encode more complex associations than just a perception of the received reward (Schultz, 2000; Simmons et al., 2007). Thus, the current study suggests that the NAcc Class I neurons rapidly adapt to encode correct associations between sensory stimuli and profitable behaviors.

Moreover, higher Class I neuron activity on a correct trial was associated with increased likelihood of making the correct association on subsequent presentations of the same visual cue. This was true despite other possible intervening trials. In addition, during the inter-trial period of the task, Class I neuron activity was low, but progressively increased as the trial proceeded (reaching higher rates at the go-cue, feedback, and reward periods of the task). This trial-by-trial fluctuation in firing rates is characteristic of striatal PAN's, which are thought to be medium-spiny projection neurons (Schultz et al., 1992).

In contrast, the Class II neurons responded primarily to immediate trial outcomes, and may represent local mechanisms of reinforcement. Unlike the Class I neurons, the mean firing rate of Class II neurons was relatively consistent across the various epochs of the behavioral task, and was only different at the reward periods when trial outcomes were revealed. This responsiveness to reward is consistent with previously reported activity of cholinergic interneurons (TAN's) of the ventral striatum (Morris et al., 2004; Apicella, 2007). Of note, individual Class II neurons also modulated relative to specific epochs of a task, and thus may play a role in providing context-dependent motivational cues (Apicella, 2007). However, interpretation of these results must also include consideration of the fact that Class II neurons tended to have higher baseline firing rates than Class I neurons. This difference may skew toward detection of increases in Class I neuron firing rates during various task epochs, while increasing the probability of detecting decreases in Class II neuron activity.

Previous studies have also suggested that specific groups of NAcc neurons encode “selection and execution of specific motivated behaviors” (Taha et al., 2007). With regard to timing, learning-related activity of Class I neurons in the NAcc is prominent early in the trials (when it can most effectively influence behavior) before movement initiation. Moreover, this activity rises for novel stimuli as the association is mastered. In contrast, the activity of Class II neurons is higher at the reward periods of incorrect trials. Thus, as alternative behaviors are explored and profitable behaviors are discovered, the activity of Class II neurons diminishes, potentially enabling the incentive values of stimuli to be encoded by the Class I neurons via mechanisms of synaptic plasticity. Therefore, the distinct but complementary activity of these two different classes of NAcc neurons may underlie mechanisms involved in learning reinforcement of profitable behaviors during operant conditioning.

## Materials and methods

### Animal model

The current study was conducted in strict accordance with guidelines set by the National Institutes of Health and protocols approved by the Animal Review Committee at Massachusetts General Hospital. Prior to starting the study, a titanium head post and standard plastic recording chamber (Crist Instrument Co.; Bethesda, MD) was surgically implanted on each primate. The chamber position was calculated based on magnetic resonance (MR) images (1.5 tesla) referenced to stereotactic atlas coordinates (Paxinos et al., 2000). Post-operatively, the animals were re-scanned to verify chamber placement. In order to verify chamber placement, fiducial markers (glass rods filled with vitamin E) were inserted into the recording chamber at known locations (Figures 1B,C). The known distance between rods was used to scale MR images and to correct for distortions. Projected trajectories for the recording chambers were then calculated using the OsiriX DICOM viewer (http://www.osirix-viewer.com/). An example of projected recording trajectories from NAcc is illustrated in Figure 1B. The imaging data along with electrophysiological mapping data (described in the subsequent section) were used to define the borders of the NAcc.

### Single-unit recording and localization of NAcc

Single microelectrodes (300–500 kOhm impedance at 1 KHz; FHC, Bowdoinham, ME) were inserted into the NAcc through grid holes spaced at 1 mm intervals using a microelectrode manipulator (David Kopf Instruments; Tujunga, CA) mounted to the recording chamber. Prior to data collection, the borders of the NAcc were electrophysiologically mapped. Neurons in the NAcc are characterized by relatively low firing rates (2–15 spikes/s), and contain neurons with regular firing rate patterns (described as TAN's) as well as neurons that fire phasically relative to behavior (PANs). The recording trajectories in the current study began in the white matter rostral to the caudate nucleus, and extended through the caudate (characterized by an increased background signal and a relative parity of neurons). From the caudate nucleus the trajectory passed into the anterior limb of the internal capsule, which was identified by a decrease in background signal with few isolatable units. As the electrode continued ventrally, groups of neurons within the NAcc were encountered. Confirmation of electrode position was achieved by locating the anterior commissure and comparing the trajectory mapping to the stereotactic atlas (Paxinos et al., 2000). Recordings in both monkeys were made from 20 to 25 mm anterior to the intra-aural point.

### Data acquisition

Analog extracellular signals were amplified and band-pass filtered at 300 Hz–6.5 KHz (Alpha-Omega Engineering; Nazareth, Israel). Behavioral and electrophysiological data were captured on a single computer acquisition system (Spike 2; Cambridge Electronic Design, UK). The analog electrophysiological and behavioral data were simultaneously digitized at 1 and 20 KHz, respectively, and were then saved for offline analysis. Electrophysiological data were sorted into individual units using an offline spike sorter (Plexon Incorporated; Dallas, TX). Autocorrelograms, spontaneous firing rates and inter-spike intervals were computed for each unit. Units with asymmetric autocorrelograms, indicating drift in their instantaneous firing rate, or an absence of refractoriness in their inter-spike intervals were excluded from further analysis.

### Learning analysis

Following recording sessions, continuous learning curves were created from the series of correct and incorrect trials. We used a state-space smoothing algorithm for point processes to estimate the point at which learning occurred (Wirth et al., 2003; Smith et al., 2004; Williams and Eskandar, 2006). This algorithm uses a Bernoulli probability model to estimate the animal's learning from their binary trial performance (0 = incorrect choice, 1 = correct choice) for each novel object. A learning criterion trial was defined as the first trial when the lower 99% confidence bound of the learning surpassed chance (25% for four possible targets). Therefore, the criterion trial represents the estimated point at which the animal learned the association. Only novel objects reaching this criterion were included in subsequent analyses. Because novel objects were learned at different rates, behavioral, and neuronal data were aligned to the criterion trial (defined as trial zero) to evaluate changes in activity during comparable phases of learning (Wirth et al., 2003; Williams and Eskandar, 2006). Because learning did not occur for familiar objects, alignment to criterion was not applicable.

### Neuronal classification

Neurons were classified by comparing firing rates during the inter-trial baseline period (500 ms before the start of the trial) to neuronal activity during the trial. Neurons were classified as responsive if their firing rate statistically modulated relative to their inter-trial baseline firing rates during one or more epochs of the behavioral task (comparisons of 500 ms of neuronal activity at the onset of each epoch; Wilcox rank-sum, *p* < 0.05). Comparisons between baseline and intra-trial activities were quantified in familiar objects trials. Only neurons that significantly modulated during the task were used for subsequent analyses.

The neurons were further subdivided into two groups based on their correlation to the learning curve. Each neuron was classified as either positively or negatively correlated (correlation coefficients being greater than or less than zero, respectively) to the learning curve during the reward epoch of the task. Each classification was then pooled, and aggregate responses were assessed. To facilitate comparison between neurons, firing rates were normalized by subtracting the firing rate for each epoch by the minimum rate for all epochs, and then dividing the difference by the range of the firing rates (maximum minus minimum for all epochs). Hence, the normalized firing rates for all neurons fell between zero and one.

### Statistical analysis

Statistical significance was evaluated by comparing firing rates of novel and familiar objects relative to learning for each task epoch (i.e., start, stimulus, go-cue, feedback, and reward periods). Novel-to-novel and novel-to-familiar comparisons were made by performing Friedman repeated measures analysis of variance with a Dunn's multiple-comparison correction. Comparisons were made using novel object mean neuronal activities before (trials −10 to −7), during (trials −1 to 1), and after (trials 3–5) learning. In addition, comparisons were made using mean familiar object activities (across all trials). Learning-related activity was considered statistically significant when neuronal responses to novel and familiar objects were different relative to learning and where neuronal responses to novel objects changed over the learning period (Friedman repeated measures analysis of variance with Dunn's correction, *p* < 0.05).

ROC curves were calculated for the two responsive neuronal populations in order to determine the ability to predict the subsequent trial outcomes based on the neuronal activities. To perform this calculation, the individual normalized neuronal activities for each trail (by epoch) as well as the outcomes for the subsequent trial were stored in a database. Once the database was built, neurons were subdivided into the two responsive groups (e.g., Class I and II), and ROC curves (MatLab, Mathworks, Natick MA) were calculated for each task epoch. Significance for each ROC curve was established by bootstrap randomization of the data (1000 randomization) and calculation of the 5 and 95 confidence bounds of each signal.

## Author contributions

John T. Gale and Emad N. Eskandar conceived of the project. John T. Gale, Yumiko Ishizawa, and Donald C. Shields trained the animals on the behavioral task and collected the experimental data. John T. Gale, Emad N. Eskandar, and Donald C. Shields performed the analysis and interpretation of the data. John T. Gale, Emad N. Eskandar, Yumiko Ishizawa, and Donald C. Shields prepared the manuscript and edited the final version.

### Conflict of interest statement

The authors declare that the research was conducted in the absence of any commercial or financial relationships that could be construed as a potential conflict of interest.
